# Macrophages and microglia: the cerberus of glioblastoma

**DOI:** 10.1186/s40478-021-01156-z

**Published:** 2021-03-25

**Authors:** Alice Buonfiglioli, Dolores Hambardzumyan

**Affiliations:** 1 Department of Oncological Sciences, The Tisch Cancer Institute, Icahn School of Medicine, 1425 Madison Avenue, Room 15-20B, New York, NY 10029 USA; 2 Department of Neurosurgery, Icahn School of Medicine, Mount Sinai, New York, NY 10029 USA

**Keywords:** Glioblastoma, Macrophages, Microglia, Microenvironment, Heterogeneity

## Abstract

Glioblastoma (GBM) is the most aggressive and deadliest of the primary brain tumors, characterized by malignant growth, invasion into the brain parenchyma, and resistance to therapy. GBM is a heterogeneous disease characterized by high degrees of both inter- and intra-tumor heterogeneity. Another layer of complexity arises from the unique brain microenvironment in which GBM develops and grows. The GBM microenvironment consists of neoplastic and non-neoplastic cells. The most abundant non-neoplastic cells are those of the innate immune system, called tumor-associated macrophages (TAMs). TAMs constitute up to 40% of the tumor mass and consist of both brain-resident microglia and bone marrow-derived myeloid cells from the periphery. Although genetically stable, TAMs can change their expression profiles based upon the signals that they receive from tumor cells; therefore, heterogeneity in GBM creates heterogeneity in TAMs. By interacting with tumor cells and with the other non-neoplastic cells in the tumor microenvironment, TAMs promote tumor progression. Here, we review the origin, heterogeneity, and functional roles of TAMs. In addition, we discuss the prospects of therapeutically targeting TAMs alone or in combination with standard or newly-emerging GBM targeting therapies.

## Main

Glioblastoma (GBM) develops in the complex tumor microenvironment (TME) of the brain. The GBM TME consists of extracellular matrix (ECM), interstitial fluid, and non-neoplastic cells, including both immune and non-immune cells that are compartmentalized in anatomically-distinct regions referred to as tumor niches, where the most therapy-resistant glioma stem cells (GSCs) are localized [1]. The various non-neoplastic cells closely interact with neoplastic cells and with each other in the TME by secreting growth factors, chemokines, cytokines, ECM constituents, angiogenic molecules, and factors that induce vascular permeability, creating strong interdependence that drives tumor progression. Such strong interdependence has been demonstrated between glioma cells and the most abundant non-neoplastic immune infiltrates in the TME called tumor-associated macrophages (TAMs). TAMs support tumor proliferation, regulate immunosuppression, contribute to cerebral edema, and enhance GBM resistance to chemo- and radiotherapy (RT) [2–4]. Therefore, they have been established as viable targets for adjuvant therapy for GBM. To develop successful TAM-targeted therapies, a thorough understanding of the inter- and intra-tumoral heterogeneity and plasticity of TAMs is required.

## Current state of TAM ontology

**Fig. 1:**
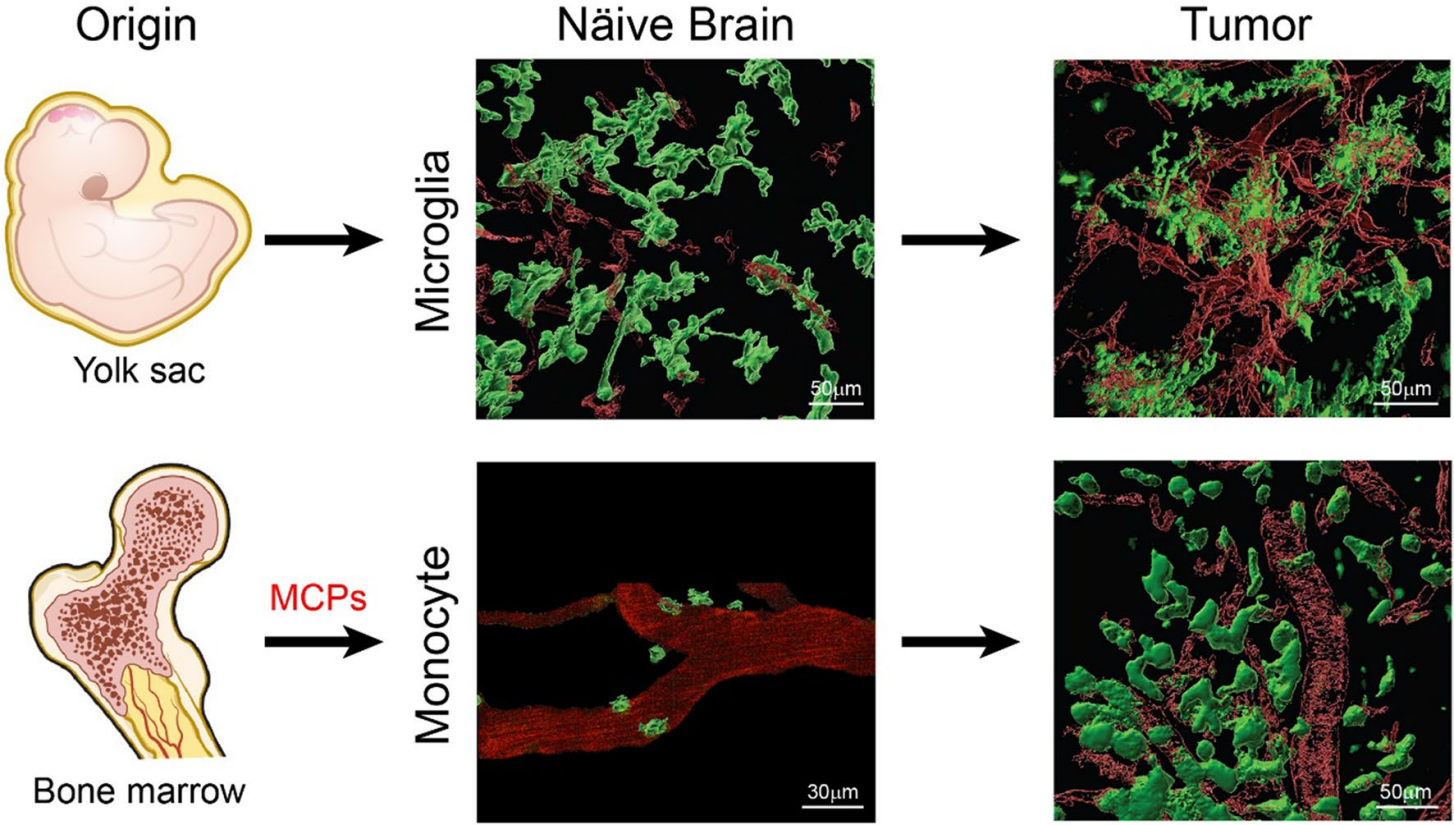
Myeloid cell lineage and morphology in healthy brain and glioblastoma. Microglia arise from yolk sac progenitors in the embryonic stage and reside permanently in the brain parenchyma. They appear as highly-ramified cells, but they rapidly change their morphology into an amoeboid-like shape in glioblastoma. Monocytes originate from the bone marrow and circulate in the blood, until they invade the brain when a glioblastoma emerges and differentiate into macrophages. They are round shaped while in circulation and upon infiltration into glioblastoma, making them morphologically indistinguishable from microglia. Images are taken from the murine glioblastoma model generated by using RCAS/tv-a, a somatic cell type-specific technology [41]. Reciprocal bone-marrow chimeras generated by using Cx3cr1-GFP mice allowed generation of only GFP-labeled bone marrow-derived macrophages or GFP-labeled microglia for imaging. Blood vessels are visualized with TRICT-dextran. Scale bars, 30 and 50 µm [41]

The most abundant non-neoplastic cells in the TME of GBM are TAMs, which constitute ~ 40% of the tumor mass [1, 4–6]. TAMs are a diverse population consisting of both brain-resident microglia and bone marrow-derived macrophages (BMDMs) (Fig. 1). Microglia is a unique myeloid population of the central nervous system (CNS). They are mononuclear phagocytic cells, which are often referred to as CNS-specific macrophages in the literature. Macrophages are known to maintain tissue homeostasis during development and in physiology; they also constitute the innate immune myeloid cells [7, 8]. Macrophages are found dispersed throughout the mammalian body. Their functions are highly plastic and depend upon the tissue in which they reside [9–12]. Microglia were shown to exclusively originate in the yolk sac from erythro-myeloid progenitor cells (EMPs) during embryogenesis [13] and to present high longevity (15 months on average) and low self-renewal [14–17]. This traditional view has been challenged by recent data, which used Ccr2-CreER for lineage tracing to demonstrate that a 25% of microglia in the brains of P2-P24 mice are derived from a hematopoietic-monocytic population [18, 19]. Details regarding the ontogenesis of various myeloid cells in the brain are provided in greater detail in a previous review [4]. Furthermore, additional new studies showed transcriptomic heterogeneity of microglia in various regions of mouse brain, suggesting the existence of a variety of microglial sub-phenotypes depending upon their topological distribution and protein expression levels in both the mouse [20] and human [21] brain. In addition, it was shown that transcriptional states of human microglia are determined by their spatial distribution, and they further change with aging and brain-tumor pathology [22, 23]. Recent fate-mapping studies also revealed the presence of non-parenchymal macrophages at CNS interfaces (otherwise called border-associated macrophages, BAMs), like meningeal and perivascular macrophages, whose ontogeny is similar to microglia [24]. BAMs are also present in the choroid plexus, where they are further classified as stromal macrophages and Kolmer’s epiplexus cells, whose ontogeny is more complex: both have been shown to originate during embryogenesis from EMPs, but only the former present high self-renewal from BM monocytes postnatally [25]. Single-cell transcriptional studies revealed that each of these macrophages have a distinct phenotype, transcriptional signature, and signaling requirements [26].

In the periphery, tissue macrophages originate from a hematopoietic stem cell (HSC) pool during embryogenesis in the fetal liver [13, 27]. At embryonic day 12.5 (E12.5), HSCs generate fetal monocytes, which present as two populations: Ly6C^+^CX3CR1^int^ and Ly6C^−^CX3CR1^high^, with both expressing the C–C chemokine receptor type 2 (CCR2) [13, 28]. Lineage tracing experiments using inducible or constitutive Cre-reporter mice demonstrated that the Ly6C^+^ population is actually a mandatory precursor of the Ly6C^−^ population, which exhibits restricted lifespan [27]. Additionally, studies have shown that the Ly6C^+^ monocytic population is the one that emigrates from the fetal liver into the blood, resulting in the downregulation of Ly6C and the initial expression of CX3CR1, which culminates in the cells infiltrating the tissue and differentiating into macrophages [7]. With few exceptions, like splenocytes [29] and skin or gut macrophages, these cells remain in the tissue postnatally with longevity and limited self-renewal [12, 30, 31].

Postnatally and during adult life, hematopoiesis takes place in the BM and also in the spleen, with the generation of Ly6C^+^ monocytes that extravasate the BM in response to monocyte chemoattractant proteins (MCPs) and circulate in the blood. In healthy conditions, monocytes have exceedingly short circulation half-lives, which are ~ 19 h for Ly6C^+^ and ~ 2.2 days for Ly6C^−^ [27], unless a pathological insult occurs, such as blood–brain barrier (BBB) disruption during a brain tumor or inflammation. In such occasions, monocytes extravasate and populate the inflamed brain tissue, where they differentiate into BMDMs [12, 32, 33].

Microglia present a highly-ramified morphology in surveillance mode, and when activated, they rapidly change their morphology into an amoeboid state [34]. BMDMs, however, are round-shaped like activated microglia, making them indistinguishable in histological sections. When lineage tracing is not used, these two cells can be discriminated by using differential expression of the CD11b/CD45 markers (CD45 is low in microglia and high in macrophages), together with the Ly6C and Ly6G markers (CD11b^+^CD45^low^Ly6C^−^Ly6G^−^ for microglia and CD11b^+^CD45^high^Ly6C^low^Ly6G^−^ for macrophages) [35]. Bowman and colleagues used genetically-engineered mouse models (GEMMs) to track the ontogeny of myeloid cells, such as the hematopoietic tracking system Flt3:Cre;Rosa26:mTmG, and were able to illustrate BMDM contribution to the TAM population in brain tumors [36]. This model allowed not only confirmation of the ontogeny of microglia and monocytes, but also definition of the immune tumor composition when combined with different glioma mouse models, such as GEMMs and GL261-cell line-based models [36]. Previous works from our laboratory used a similar approach to define the TAM composition in murine GBM GEMMs [35]. To this end, we used *Cx3cr1*^*GFP/*+^;* Ccr2*^*RFP/*+^ knock-in mice combined with a GEMM of PDGFB-driven GBM and showed that 85% of TAMs are BMDMs, which are predominantly localized in perivascular areas of the tumor, while microglia were peri-tumoral. These findings were confirmed for human GBM by using high-dimensional unbiased single-cell RNA-sequencing from fresh GBM patient samples [37]. In addition, the authors argued against *status quo* therapeutic strategies that target TAMs indiscriminately, and instead suggested favoring strategies that specifically target immunosuppressive blood-derived macrophages [37]. Using RNA-sequencing, it was revealed the different transcriptional patterns of infiltrating and resident TAMs that result in differential functions that can be manipulated for therapeutic strategies. Specifically, metabolism and pro-inflammatory cytokine-related genes are enriched in tumor-associated microglia, while cellular migration is upregulated in TAMs [35]. Various mechanisms were proposed for monocyte migration into GBM. For instance, MCP-1, and partially MCP-3, are known to promote monocyte infiltration in inflammatory conditions and glioma [38–40], where they differentiate into BMDMs. However, other MCPs that cluster on the same genomic locus, such as MCP-2 and MCP-5, cannot be excluded from playing an active and perhaps redundant role in this process, which is probably why single chemokine-targeted therapies have been unsuccessful in both blocking monocyte infiltration and treating tumors.

More recently, by performing in vivo 2-photon imaging, one study was able to define TAM morphology and localization, and to trace their infiltration and differentiation dynamics. This study also demonstrated that monocyte infiltration is not only driven by increased chemokine gradient in tumors, but it is also influenced by disruption of the BBB [41]. Two-photon imaging provides the ability to distinguish the different morphology, round for macrophages and ramified for microglia, as well as to define their temporal and spatial localizations. Novel markers unique for microglia have been proposed such as SALL1, TMEM119 or SIGLEC-H [42–44], or P2RY12/SLC2A5/FCRLS for microglia and GDA/EMILIN2/HP/SELL for macrophages [45]; so far, only the latter have been tested in mouse and their expression, although faithful in healthy CNS, changes in the context of tumors.

## Activation and heterogeneity of TAMs

As already stated above, TAMs are a heterogeneous population, based not only upon their origin and localization within the tumor, but also on their functions. Initially, upon activation, TAMs were classified into two different phenotypes: (1) a pro-inflammatory M1 phenotype/polarization, characterized by the classical activation of immune receptors TLR2/4 and the production of pro-inflammatory cytokines, including TNF and IL-1β, and (2) the anti-inflammatory M2 phenotype/ polarization, with the production of ARG1, IL-10, and IL-4 [46]. Historically, in the context of GBM, TAMs were considered to possess an M2-like phenotype [47]. However, transcriptional analyses have shown that this classification is an oversimplification of the otherwise complex biology of these cells [48]. In fact, microglia and macrophages share both M1 and M2 phenotypes in the setting of murine brain tumors [49]. For example, ARG1 and IL-1β were found to be enriched in both tumor-associated microglia and macrophages [35]. In human GBM, microglia and macrophages more resemble the expression profile of non-polarized M0 macrophages [50]. Since the challenges to discriminate microglia from macrophages in human GBM still exist, TAMs as a whole were used to characterize their intra-tumor heterogeneity. Thus, single-cell transcriptomic analysis and cytometry by time of flight (CyTOF) in human GBM and control tissues revealed that TAMs are not only heterogeneous, but they also show M1-like genes, such as SPP1, APOE, or CD74, as well as M2-like genes, including HLA-DR and CD163. Interestingly, the authors also compared this TAM phenotype to disease-associated microglia (DAMs) and found a similar transcriptional spectrum to neurodegenerative and neuroinflammatory diseases [23]. A recent meta-analysis of available single-cell and bulk RNA sequencing of human patient GBM samples suggests a dynamic identity (with the presence of M0, M1, and M2 states) of TAMs, with a more pro-inflammatory phenotype in the tumor core versus a more anti-inflammatory state in the periphery. Interestingly, this study also proposed specific region-associated functions of TAMs: an increased activity of the PD-1 signaling pathway was observed in the tumor core, versus stronger NF-kB signaling in the tumor periphery [51]. The studies mentioned above clearly illustrate that the simple M1/M2 dichotomy is no longer applicable and should not be used in GBM.

Temporal and spatial localization are additional differences shared by tumor-associated microglia and macrophages. Microglia are found to be prominent in the peri-tumoral areas, while macrophages are dispersed inside the tumor bulk, more in the perivascular area [35, 41]. Lineage tracing experiments using GEMMs showed early infiltration of tumor-associated monocytes and their differentiation into tissue macrophages [35]. Migratory capacity is yet another parameter in which they differ: monocytes are highly motile and display both straight and random movements, but upon tumor entry, they rapidly differentiate into macrophages and display limited movement, which may explain their localization in close proximity to blood vessels. Microglia, on the other hand, are more stationary, as they extend and retract their processes and have larger mean surface area and higher numbers of branches when compared to macrophages, which may contribute to their localization in peri-tumoral areas [41]. Recent high-resolution single-cell RNA-sequencing (scRNA-seq) in both human and murine brain tissue has provided evidence for high heterogeneity within microglial populations in healthy conditions [22, 23, 52], so it will be interesting to see whether these regional and age-dependent heterogeneities existing in healthy brain will have a similar impact in the context of brain tumors.

Gene expression analyses have started characterizing TAM composition, and subsequently their functions, in different GBM subtypes, with the aim of finding better therapies. By using nanostring analysis, human-established GBM subtypes (Proneural (PN), Mesenchymal (MES), and Classical (CL), see BOX 1) were examined for expression of *AIF1* (encoding IBA1) as a marker for TAMs. It was found that AIF1 expression is higher in MES GBM compared to PN and CL. Additionally, *AIF1* expression was found to influence the survival rate with regard to the subtype analyzed: higher *AIF1* levels in MES are associated with longer survival, while in PN tumors survival is shorter when *AIF1* expression is high [53]. This was consistent with previous studies that additionally related higher TAM infiltration to the *NF1* gene mutation [54, 55]. In addition, Bhat and colleagues proposed that PN tumors transitioning into MES are associated with increased TAMs and activation of the TNF/NF-κb signaling pathway [56]. Similarly, due to TAM inter- and intra-tumoral heterogeneity, specific signaling important for TAMs in one model may not be relevant in other models. A classic example is CSF1R inhibition, which caused TAM reduction in other solid tumors [57, 58] and resulted in the elimination of ~ 95% of microglia in naïve mice [59], and while proving effective in targeting TAMs in PDGFB-driven adult glioma mouse models, it was not effective in reducing their number, but instead it changed their expression profile [60]. These data suggest that while CSF1R is not important for TAM survival in PDGFB-driven tumors, its importance for TAMs in other subtypes remains to be determined, especially in light of the recent data showing that various driver mutations in adult GBM models can create different numbers, expression profiles, and compositions of TAMs, similar to what is observed in human GBM [50, 53, 55]. In addition, it was recently demonstrated that in the most common malignant pediatric brain tumor, medulloblastoma, CSF1R has an anti-tumoral affect, and targeting it resulted in accelerated tumor growth [61]. Whether these differing results reflect GBM subtype-specific responses to blocking CSF1R^+^ TAMs, as observed in recent GBM anti-angiogenic trials [62], remains to be determined. A recent study used an integrated panel of RNA-seq, flow cytometry, protein arrays, and culture assays to characterize the differences in TMEs, specifically in immune composition and myeloid cells in primary and metastatic brain tumors [63]. It would be interesting to see how the conclusions from this study would be affected by analysis of the use of dexamethasone (DEX), since the vast majority of patients with both primary and metastatic brain tumors develop vasogenic edema, which is managed primarily with DEX. Whether DEX was used in the so-called “control brain” samples is another critical question [63]. In light of recent evidence showing that DEX targets both adaptive and major inflammatory pathways in tumor-associated myeloid cells in GBM, caution should be exercised in the interpretation of immune profiling studies from both fresh patient primary and metastatic brain-tumor samples [3]. Using GBM GEMMs that are based upon human GBM-specific driver mutations, it was shown that TAM numbers, compositions, and expression profiles are dictated by driver mutations, further highlighting the intra- and inter-tumor heterogeneity of these cells [41, 48, 64], similar to what was shown in human GBM subtypes [50, 53, 55]. Isocitrate dehydrogenase (IDH) mutations (mainly in the IDH1 and IDH2 genes) have been shown to influence immune TME behaviour in both low- and high-grade gliomas [65–68]. For instance, Berghoff et al. showed that IDH mutation in GBM patients leads to less lymphocyte infiltration and lower PD-L1 expression, partly due to different levels of methylation of the PD-L1 gene promoter [65]. By using in vitro and in vivo glioma models, Bunse and colleagues showed that the glioma cell-derived oncometabolite (R)-2-hydrixyglutarate (R-2HG) is able to suppress CD8^+^ cell activity by interfering with the calcium-dependent transcriptional activity of nuclear factor of activated T cells (NFAT) and inhibiting T-cell receptor (TCR) signalling [66]. Mutated IDH1 reduces leukocyte chemotaxis in a murine IDH1-mutated GBM model [68]. In low-grade gliomas (LGGs), IDH1 mutation in a murine model reduced T-cell infiltration and decreased the expression levels of CXCL10 and its regulator STAT1 [67]. In vitro studies also showed that IDH1 mutation-derived transcriptional silencing of the ligand NKG2DL led to NK cell tolerance toward the tumor and lack of tumor-cell lysis by the innate immune cells [69].

Overall, these results support the concept of distinct and molecularly heterogeneous TAMs in GBM subtypes, warranting further investigation into the biological relevance of TAM heterogeneity in various GBM subtypes. In addition, studies have clearly demonstrated that various driver mutations have significant impacts on both TAM function and composition. The redundant approach of studying TAMs in various GBM subtypes will help us to better define common TAM-derived mechanisms that can be pan-GBM targets and/or to identify unique TAM targets for a given subtype.

## TAMs promote tumor progression

**Fig. 2:**
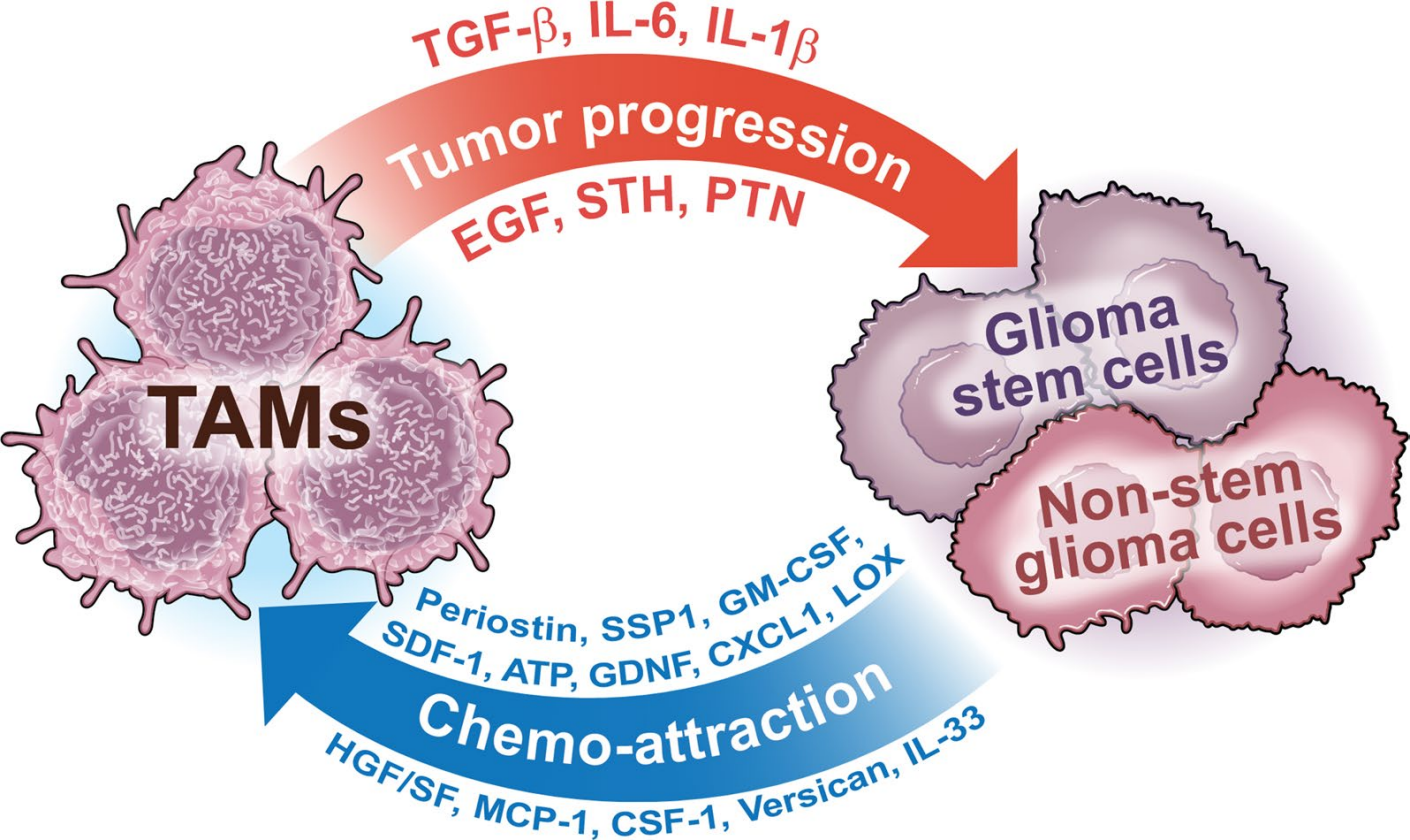
Tumor-associated macrophage (TAM)-glioma cell crosstalk creates a strong interdependence that leads to increased TAM recruitment, which supports tumor growth. Glioma cells—including both glioma non-stem cells and glioma stem cells—release chemoattractant molecules (Periostin, SSP1, GM-CSF, SDF-1, ATP, GDNF, CX3CL1, LOX, HGF/SF, MCP-1, MCP-3, CSF-1, Versican, and IL-33) that recruit and reprogram TAMs. In turn, TAMs release factors (IL-6, IL-1β, EGF, TGF-β, STI1 and PTN) that promote tumor progression

It is now clear that TAMs play a major role in supporting glioma growth, invasion, and survival. It is still under debate, however, whether the tumor cells first reprogram the immune cells to a pro-tumorigenic phenotype, or vice versa, if the immune cells directly induce tumor growth. Nonetheless, the crosstalk between tumor cells and TAMs is clear but complex (Fig. 2).

TAMs have been shown to release a plethora of factors that stimulate tumor growth and invasion, including TGF-β, IL-1β, IL-6, stress-inducible protein 1 (STI1), and epidermal growth factor (EGF) by acting on GSCs in the perivascular niche and glioma cells. TGF-β, for instance, is secreted by TAMs and causes the production of matrix metalloproteinase 9 (MMP9), which disrupts the ECM and thus increases invasion of GSCs [70]. IL-1β overexpression in TAMs leads to p38 MAPK pathway activation and CCL2 (or MCP-1) production, which promote GSC proliferation [71].

In addition, TAM-induced release of STI1 and EGF promotes GBM growth and invasion [72, 73]. TAMs have been shown to increase the expression of PDGFRB on glioma cells, which stimulates their migratory capacity and consequently tumor progression [74]. Loss of osteopontin (SPP1) in TAMs, but not glioma cells, enhances tumor progression [75]. TGF-β2 expressed by TAMs induces MMP-2 expression and blocks the tissue-inhibitor metalloproteinases 2 (TIMP-2), promoting tumor invasion [76]. CCL8 (or MCP-2) was found to be highly expressed by TAMs and to be correlated with poor survival. In a murine GBM model, CCL8 was shown to promote GBM invasion and to increase tumor cell stemness via the ERK1/2 signaling pathway [77].

As innate immune cells, TAMs express Toll-like receptors (TLRs), which are pathogen recognition receptors that have active roles in tumor growth. For instance, expression of TLR2 is increased in TAMs, and its ablation results in better survival [78]. TLR2 promotes TAM production of MMP14, which is essential for MMP2 release and glioma invasion, and the microglial expression of the metalloprotease MT1-MMP, which cleaves the pro-MMP2 into its active form, resulting in tumor invasion [79, 80]. TLR2 has also been found to regulate MMP9 production by microglia to promote glioma growth and invasion [81]. Another TLR that plays a role in GBM is TLR4, which regulates TAM IL-6 secretion, resulting in increased GSC proliferation [82].

Additionally, TAMs express aryl hydrocarbon receptor (AHR), which has been shown to have autocrine and paracrine effects. In fact, AHR promotes CCR2 expression in TAMs and drives their infiltration into the tumor site, as well as CD39 expression, promoting CD8 T-cell dysfunction [83, 84]. At the same time, tumor cells release chemoattractant proteins that recruit TAMs, such as CCL2, CSF-1, granulocyte–macrophage colony stimulating factor (GM-CSF), hepatocyte growth factor or scatter factor (HGF/SF), stroma-derived factor 1 (SDF-1), and glial cell-derived neurotrophic factor (GDNF). GBM cells express high levels of CCL2, which recruits TAMs and promotes tumor growth [35]. However, other studies showed that CCL7 (or MCP-3), rather than CCL2, acts as a TAM chemoattractant [40]. Glioma-derived IL-33, in its secreted form, is found to be associated with TAM recruitment and infiltration [85]. Also, CSF1 (alternatively called M-CSF) promotes TAM motility and has been shown to induce the switch to a more immunosuppressive phenotype (see above [72, 86]). GM-CSF and lysyl oxidase (LOX) secreted by glioma cells promote TAM recruitment [86, 87]. However, LOX is secreted only when GBM lacks PTEN expression [87]. The role of CX3CR1 in GBM is more controversial, which can be partially attributed to the different models used in various studies. *Cx3cr1* deletion was shown to have no impact on TAM recruitment and survival of tumor-bearing mice [88], while our previous studies showed that *Cx3cr1* loss, indeed, did not affect tumor-associated microglial migration in the peritumoral areas, but increased infiltration in the perivascular areas by upregulating IL-1β expression, resulting in increased CCL2 expression in a PN GBM murine model [71]. Glioma-derived HGF/SF is postulated to be a potent chemoattractant acting upon the tyrosine kinase receptor (c-MET) expressed by monocytes and microglia [89]. SDF-1 and GDNF are other proposed factors that have been shown to promote TAM accumulation inside the tumor [90, 91]. SPP1 released by tumor cells increased TAM infiltration and immunosuppression [92]. Periostin produced by GSCs was responsible for BMDM recruitment into the perivascular niche and enhanced glioma progression [93].

Recent endogenous ligand screenings have revealed several new molecules released by glioma cells that interact with TLRs in TAMs and promote tumor growth. Among them, Versican promotes tumor expansion via TLR2 and upregulation of MT1-MMP and MMP9 expression [78]. The cell adhesion molecule Tenascin C (TNC) has been shown to positively regulate tumor proliferation via TAMs [94]. However, very recently, it was shown that TNC increases glioma cell phagocytosis when CD47 surface marker is lost in a GBM xenograft mouse model, and the authors correlated the increased TNC levels to a potential defensive mechanism by the immune system against the tumor [95]. In addition, multiple novel mechanisms were identified specific to GSC-TAM interactions. For instance, pleiotrophin (PTN) secreted by TAMs binds to PTPRZ1 on GSCs, resulting in their activation and consequently tumor growth [96]. Additionally, GSC-secreted WISP1 has been shown to support the pro-tumorigenic phenotype of TAMs by promoting the survival of both GSCs and TAMs [97].

TAMs contribute to tumor survival against radio- and chemotherapies by initiating regenerative programs, such as wound healing, supporting tumor relapse; however, the molecular mechanisms underlying these programs are still under investigation [4, 98, 99]. The hypoxia-inducible factor 1 (HIF-1) signalling pathway has been proposed to be involved in resistance to RT; its radio-induced expression is responsible for the activation of SDF-1 and its cognate receptor CXCR4 [100], leading to monocyte recruitment as well as angiogenesis and tumor recurrence [101]. RT has the capacity to increase the recruitment of monocytes via CSF-1 [102]. Inhibition of CSF-1R enhances the efficacy of the multi-targeted kinase inhibitors vatalanib and dovitinib in a PN mouse model of GBM [103].

All of the studies mentioned above were performed using various murine and human GBM lines and models, without considering the genetic makeup of the tumors, making it difficult to determine whether each identified target is a pan-TAM target or is specific for a given model. Understanding this is vital in light of recent examples of specific TAM targeting therapies such as through CSF1R inhibition, which has proven to be extremely effective in targeting TAMs in a PDGFB-driven adult glioma model [60], but failed to demonstrate effectiveness in a clinical trial with unselected adult recurrent GBM patients [104]. Additionally, we have recently shown that CSF1R has anti-tumoral effects, and targeting it results in accelerated tumor growth in the most common malignant pediatric brain tumor, medulloblastoma [61].

In addition, in all of the studies mentioned above, there were no assessments as to whether the effects driven by TAMs were due to microglia and/or macrophages, which is very important considering that targeting monocytes in the circulation will bypass BBB limitations associated with microglia-targeted therapies.

## TAMs and immunosuppression

Immune surveillance is hampered in GBM, most likely due to multiple mechanisms. Impaired phagocytosis has been proposed as one such mechanism. Glioma cells induce the expression of the surface protein CD47 (“don’t eat me” signal), which inhibits TAM phagocytic activity [105].

The signal transducer and activator of transcription factor 3 (STAT3) signalling pathway is upregulated in TAMs and is responsible for the production of tumor-promoting inflammatory molecules, such as IL-10 and IL-6, as well as inhibition of the tumor-suppressive inflammatory molecule TNF [106]. Upregulated expression of STAT3 has been found to be mediated by the interaction between the GBM molecule S100 calcium binding protein B (S100B) and the receptor for advanced glycation end products (RAGE) expressed on TAMs [107].

One emerging hypothesis explaining the immunosuppressed TME in GBM is that TAMs convert tumor-infiltrating T cells from active to ineffective via regulation of immune checkpoints. A contributing factor to this hypothesis is the fact that TAMs constitute up to 40% of the tumor mass in both human and mouse models, in contrast to low levels of T-cell infiltration [50, 71, 108]. Immune checkpoints are negative regulatory pathways that function to inhibit T-cell activation and proliferation, therefore maintaining self-tolerance and controlling the duration and degree of the immune response. Among the immune checkpoints, the most relevant are the cytotoxic T-lymphocyte-associated antigen 4 (CTLA-4), the programmed cell death-1 (PD-1), the Fas receptor, and T-cell immunoglobulin and mucin domain-containing protein 3 (TIM3) [109].

CTLA-4 is usually expressed by activated T cells, and its role is to decrease T-cell responsiveness, preventing autoimmunity [109]. The co-stimulatory molecules CD80/CD86, expressed by TAMs, interact with CTLA-4, causing reduced T-cell activation [110, 111]. PD-1 is highly expressed by T_reg_ cells, whose function is to inhibit T-cell activation and its cytotoxic activity [112–114]. TAMs promote T_reg_ activity by binding to PD-1 via PD-L1 [106]. The Fas receptor, expressed on CD8^+^ T cells, binds to the Fas ligand expressed by TAMs, and their interaction induces lymphocytic apoptosis [115]. CD8^+^ T cells also express TIM3, whose ligand is galectin 9 expressed by TAMs, resulting in the inhibition of antitumoral T helper 1 cell response [116].

Despite emerging hypotheses that TAMs are immunosuppressive, there are no studies providing direct evidence supporting these assumptions. These hypotheses are mainly based upon the observation that TAMs express high levels of PD-L1 [117]. Moreover, a recent study showed that PD-1 is also expressed by TAMs in human and in a mouse model of colon cancer, and if targeted with inhibitors in vivo, it can increase tumor cell phagocytosis and consequently reduce tumor growth [118]. Thus, inhibition of PD-1 having an impact on TAMs in GBM cannot be excluded. A lack of systematic studies to test the role of TAMs in resistance to checkpoint blockade can be partially attributed to the lack of PD-L1 conditional knockout mice, the inability to inhibit monocyte infiltration from the blood circulation to tumors, and the lack of appropriate pre-clinical testing of this hypothesis using immunocompetent GEMMs of primary GBM. Immune checkpoint inhibition (ICI) resulted in an unprecedented response rate in many cancer types, including cancers in advanced metastatic stages such as melanoma and non-small cell lung cancer. Even though the data on the expression of PD-1/PD-L1 in GBM patients are largely correlative based upon immunohistochemical (IHC) antibody staining or data mining from TCGA [119–123], late-phase clinical trials testing ICI in randomized clinical trials in unselected recurrent GBM patients did not meet the primary endpoint of improved overall survival [124]. Genomic and transcriptomic analysis after treatment with a PD-1 inhibitor showed an immunosuppressive expression signature in non-responder patients [125]. To that end, studies have started focusing on the use of PD-1 inhibitors as neoadjuvants in GBM therapy [126, 127]. As nivolumab and pembrolizumab have shown successful outcomes when administered as adjuvants in melanoma [128, 129], researchers aimed at looking in this direction for GBM. Neoadjuvant administration of pembrolizumab, with continued adjuvant therapy post-surgery, has been shown to increase overall patient survival and to enhance local and systemic immune response in recurrent GBM patients [126]. A single-arm Phase II clinical trial of neoadjuvant nivolumab showed increased anti-tumor immune response, although limited sample size prevented drawing definite conclusion on clinical efficacy [127]. While one study did not address whether immune status may be influenced by anti-edema therapy (80% of patients enrolled in the study were currently on steroids) [127], another found no significant correlation between DEX and immune activation, while not excluding the fact that the study protocol, which excluded patients on high-dose DEX, might have contributed to the lack of correlation [126]. Unfortunately, this has not been investigated with regard to TAM functions, and it would be interesting to determine whether a neoadjuvant anti-PD-1 therapy might also boost the inflammatory response of TAMs. More recently, a subset of myeloid cells called myeloid-derived suppressor cells (MDSCs) have emerged as tumor-promoting cells, whose function is to suppress the T-cell immune response. MDSCs include a small (5% and 8% of the total myeloid population in human and mouse gliomas, respectively [130]) group of myeloid progenitors existing in three subtypes: polymorphonuclear PMN-MDSCs, monocytic M-MDSCs, and early-stage eMDSCs [130, 131]. The first two subsets present specific markers for identification, both in human and in mouse, while the eMDSCs still need to be identified and studied. PMN-MDSCs, defined by CD11b^+^CD14^−^CD33^+^HLA-DR^−^ in human and by Cd11b^+^Ly6C^low^Ly6G^+^ in mouse, are morphologically and phenotypically similar to neutrophils, while M-MDSCs, defined by CD11b^+^CD14^+^ CD33^+^HLA-DR^−^ in human and by CD11b^+^Ly6C^high^Ly6G^−^ in mouse, are more similar to monocytes and are distinguishable only by the lack expression of MHC II [132]. The basic function of MDSCs is to suppress mainly T-cell function, and to a lesser extent NK cells and B cells [132, 133]. In the context of GBM, MDSCs have been defined as myeloid progenitors in the blood (subsets of monocytes or neutrophils) that gain immunosuppressive properties in tumor settings, and when infiltrating into tumors, they can differentiate into BMDMs and neutrophils that exhibit immunosuppressive properties. In fact, they are the source of GBM-infiltrating immunosuppressive BMDMs, since there are no macrophages in circulation [4, 5, 48, 134]. In GBM patients, elevated levels of MDSCs are found in peripheral blood, but also in the tumor tissue. The types of MDSCs differ in tumor tissue compared to peripheral blood, but the composition and the subtype specificity are still not completely clear [130, 135–137]. In addition, Bayik et al. suggested that the monocytic MDSCs (mMDSCs)—CD45^+^CD11b^+^Ly6C^+^Ly6G^−^I-A/I-E- were enriched in the TME of male tumor-bearing animals, while granulocytic MDSCs (gMDSCs)—CD45^+^CD11b^+^Ly6C^+^Ly6G^−^ were enriched in the TME of female tumor-bearing animals, suggesting that MDSC subsets drive immunosuppression in a sex-dependent manner [137]. Whether the differential recruitment of subsets of MDSCs in female and male recipient animals is a result of the fact that the GL261 cell line is derived from male C3H mice [138–140] needs to be determined experimentally. In addition, the authors’ definition of gMDSCs is based upon multi-parameter FACS staining for CD45^+^CD11b^+^Ly6C^−^Ly6G^+^, and this combination defines neutrophils and does not provide evidence as to whether these cells are immunosuppressive. Recently, elegant work has provided the field with the technology to isolate and test whether myeloid cells in tumors are in fact immunosuppressive, and in the absence of performing these functional assays, the field should use caution in referring to myeloid cells as MDSCs [141]. Nevertheless, these findings are provocative and will need to be validated by using a larger panel of cell lines and GEMMs that do not require transplantation of donor tumor cells from one sex into recipients of a different sex. Recent studies have suggested that several factors, released by glioma cells but not exclusively, can induce the proliferation, differentiation, and recruitment of MDSCs, such as IL-6, GM-CSF, IL-10, prostaglandin-E2 (PGE2), VEGF, and TGF-β [131]. Another suggested regulator for MDSCs is accumulation of CCL2, which when released by glioma cells recruits CCR2-expressing MDSCs in addition to activating T_regs_ [142]. In summary, multiple subsets of MDSCs have been identified, and in many cases, they are defined based upon marker expression rather than by demonstration of their immunosuppressive function. Nevertheless, these studies clearly illustrate the existence of TAM and TAN subsets with immunosuppressive properties. We have not yet succeeded in depleting TAMs (neither microglia nor macrophages) in GBM; therefore, there may be TAM subsets that promote tumor growth and immunosuppression, while other subsets may function to restrain tumor growth. This has now been shown to be the case in pancreatic ductal adenocarcinoma (PDAC). For example, while numerous studies have highlighted the tumor-supportive functions of stromal cancer-associated fibroblasts (CAFs) in PDAC TME, recent work has demonstrated that stromal components may also function to restrain PDAC growth, highlighting the importance of functional heterogeneity within these cells [143–145]. Additionally, in a mouse model of pediatric medulloblastoma, TAMs have been proposed to have anti-tumor functions, as reduction of their infiltration decreases the overall survival time of tumor bearing-mice [61]. While there is no doubt that TAMs can be reprogrammed by the use of targeted therapies from pro-tumorigenic to an anti-tumor phenotype, to date there are no studies demonstrating intrinsic anti-tumoral functions of TAMs in GBM. However, given their anti-tumoral potential, we expect that in the near future, this picture might change.

## TAMs in GBM-associated vasogenic cerebral edema

Vasogenic cerebral edema is a common and serious complication that occurs in the majority of GBM patients as a direct consequence of enhanced vascular permeability of the BBB, leading to extracellular fluid accumulation. This eventually leads reduced cerebral blood flow, hypoxia, and increased intracranial pressure (ICP), ultimately culminating in neuronal impairments and death [146]. Despite its frequency and severity, very little is known about how edema forms. Growth factors, as well as channel and matrix metalloproteinases, are implicated in brain injury-related edema [147]. The vascular endothelial growth factor (VEGF), expressed by gliomas but also by stromal cells such as astrocytes and TAMs, is known to promote angiogenesis and tumor malignancy, and to enhance BBB permeability in brain tumors [148]. The water channel Aquaporin-4 (AQ4) is expressed by astrocytes and is suggested to play an important role in VEGFA-induced GBM-associated edema [149, 150]. MMP9, expressed by endothelial cells and astrocytes, has been shown to regulate and promote BBB disruption and cerebral edema in a rat injury model [151, 152] and it has also been associated with edema in brain tumors [153, 154].

GBM-associated vasogenic cerebral edema is almost exclusively managed with the steroid DEX. While the clinical benefits of DEX can be significant, long-term use of even low doses has significant toxicities, including diabetes, myopathy, depression, and occasionally frank psychosis [155, 156]. It has also been shown that DEX compromises the efficacy of RT in GBM [156]. Furthermore, the immunosuppressant effects of DEX counteract the efficacy of several emerging immunotherapies for treating GBM [157]. DEX decreases the numbers of infiltrating CD4^+^ and CD8^+^ T cells [158], in addition to lowering the numbers of myeloid and NK cells [159]. Recently, Iorgulescu et al. showed that DEX therapy in GBM patients reduces patient survival in a dose-dependent manner and increases the resistance to ICI [159].

Although anti-VEGFA therapy is initially effective at decreasing edema at high anti-cancer doses, GBM rapidly adapts to it, leading to rapid tumor progression without improvement in overall survival [160–162]. Radiographic and tissue studies of patients with GBM who were treated with Avastin or Cediranib (a small-molecule VEGFR inhibitor) support the results of enhanced tumor invasiveness and increased infiltration of tumor-promoting TAMs and other CD11b^+^ myeloid cells [163], similar to what was also observed in a PN mouse GBM model [156]. More studies are needed to determine the effects of anti-VEGFA treatment on other immune cells and to determine whether it interferes with emerging novel immunotherapies in GBM. Clearly, new approaches for effectively treating GBM-associated edema are needed, which ideally should target the driving mechanisms of edema formation in GBM and be devoid of the deleterious effects typical of DEX and anti-VEGF-A therapy.

Historically, myeloid cells have not been known to play an integral role in BBB maintenance in the healthy brain. However, due to their perivascular localization in GBM, TAMs became of particular interest because of a recent study evaluating the mechanism driving the anti-edema effects of DEX. This study established that DEX inhibits TAM production of the pro-inflammatory cytokine IL-1β [3].

**Fig. 3:**
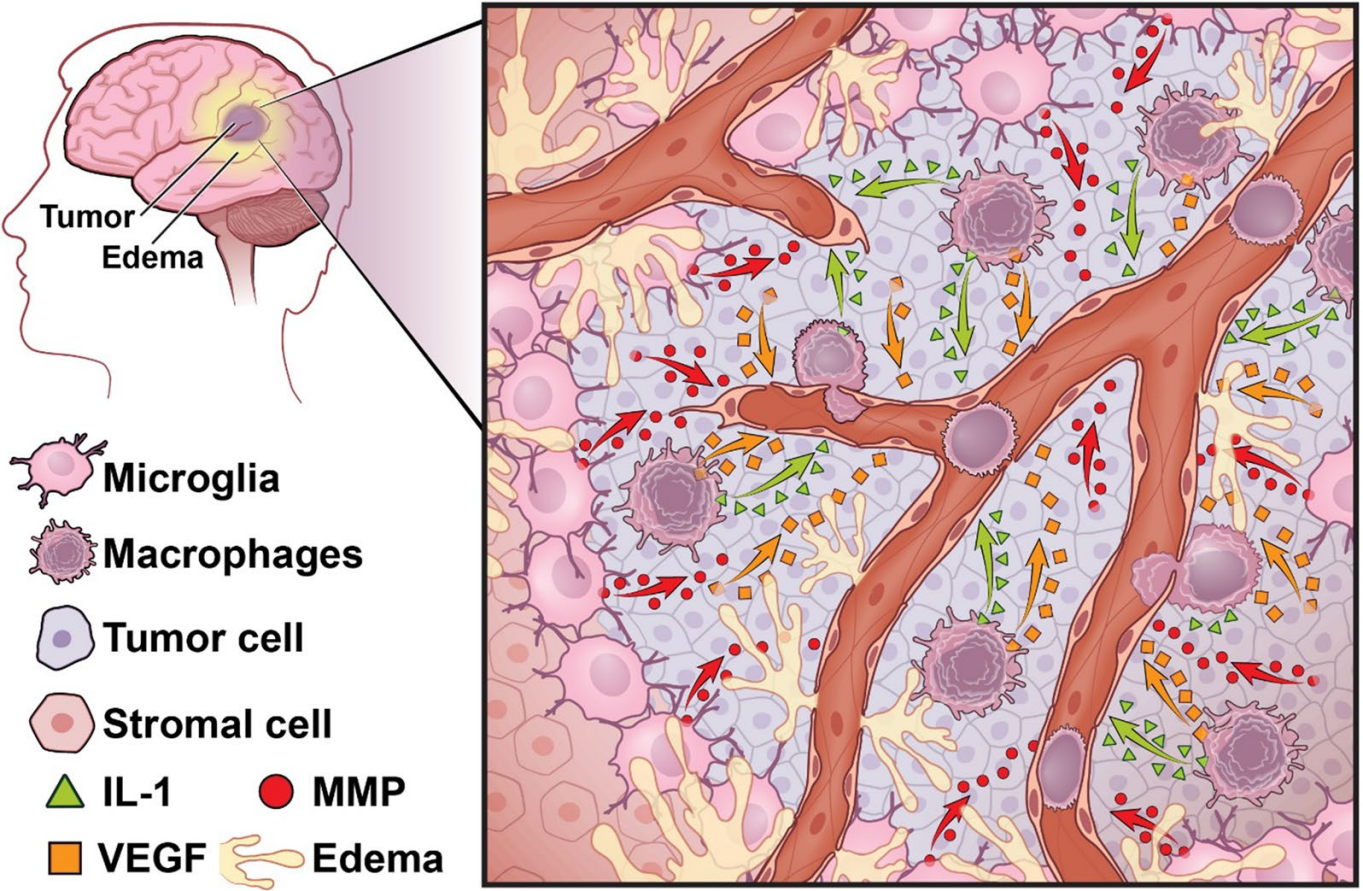
Vasogenic cerebral edema in glioblastoma. Schematic illustration of the cell populations and soluble factors that regulate glioblastoma-associated vasogenic cerebral edema in the perivascular area. Magenta color indicates the tumor, while yellow color shows edema surrounding the tumor. Edema causes additional mass effects, often exceeding the mass induced by the tumor itself. When secreted in the perivascular areas, IL-1β [3] and MMPs [79] produced by TAMs, and VEGFA [166] produced by TAMs and tumor cells, increase vascular permeability and edema formation

When *Il-1α/β* or *Il-1β* were genetically ablated in a mouse model of PN GBM, magnetic resonance imaging (MRI) as well as histological analyses revealed decreased cerebral edema comparable to levels achieved by DEX or anti-VEGFA treatment, suggesting that inhibition of the IL-1β-mediated pro-inflammatory pathway may be a promising alternative therapy for cerebral edema [3]. These findings are supported by the work of Sehm and colleagues, who administered Sulfasalazine, a potential inhibitor of IL-1β production in macrophages [164], and showed reduced cerebral edema in a rat glioma model [165]. These initial findings are only the first steps in a much longer journey to uncover the important role that TAMs play in the formation of GBM-associated vasogenic cerebral edema, and they also encourage extension of these investigations into further understanding the complex interactions between TAMs and endothelial cells, pericytes, and astrocytes in the perivascular area (Fig. 3).

In addition, the potential role of other inflammatory cytokines, such as IL-6 and TNF, may be worthwhile to investigate, as they have been shown to regulate the expression of integrin β4/α5 subunits and α2, respectively [167, 168] and may alter BBB permeability. Furthermore, it would be interesting to investigate whether other MCPs, such as CCL-2 or its cognate receptor CCR2, play a role in regulating cerebral edema diffusion. Interestingly, the genetic loss of *Ccr2* in a mouse model of ischemia was able to decrease vascular permeability and cerebral edema [169].

## TAM interactions with the TME

In addition to the ability of TAMs to suppress T-cell function, interesting and novel TAM—T cell interactions in TME have been documented in LGGs. For example, recent studies in LGGs demonstrated that microglia/T-cell interactions are reciprocal and are essential for Nf1 optic glioma growth [170, 171]. Additionally, it has been shown that neurons produce midkine, which in turn activates T cells to produce CCL4, which induces the microglial production of CCL5, which is essential for promoting Nf1 optic glioma [172]. This study elegantly illustrates that microglia promote tumor growth not only by directly interacting with tumor cells, but also by establishing complex interactions with other cell types in the TME, in this case via the neuron/T-cell/microglia axis [172]. While these studies are performed in LGGs, it would be interesting to evaluate these interactions in GBM. Increasing evidence suggests that neutrophils, which are an integral part of the innate immune system, also play a pro-tumorigenic role in GBM, and they are often referred to as tumor-associated neutrophils (TANs) [173, 174]. Neutrophil infiltration and activation are considered to be markers of poor glioma prognosis. Increased neutrophil degranulation, elevated levels of ARG1 that suppress T-cell functions, upregulation of S100A4 expression, and increased IL-12 levels have been shown to be associated with glioma malignancy [175, 176]. In addition, depletion of TANs by using a monoclonal antibody against Ly6G resulted in increased survival of an IDH wild-type (WT) glioma mouse model [176]. Interestingly, IDH MUT, which is less aggressive than the IDH WT glioma, has low tumor TAN infiltration and it is correlated to the downregulation of chemotaxis-related genes [68]. Similarly, the most aggressive GBM subtype, MES GBM, has higher neutrophil infiltration [55]. A few studies have investigated the interactions between TAN and glioma cells, and identified IL-6 and IL-8 as tumor-secreted key factors for TAN activation [177]. However, the mechanism of neutrophil recruitment, and their interplay with TAMs in GBM growth, are still unknown. However, monocytes and neutrophils are considered to be “partners in crime”; e.g., they coordinate an effective immune response. For example, in response to microbial challenge, tissue-resident macrophages produce neutrophil chemoattractants such as CXCL1, CXCL2, and IL-1α, which results in rapid recruitment of neutrophils to the site of infection [178]. It was recently shown that neutrophils infiltrate tumors and induce ferroptosis, which results in increased tumor necrosis and promotes GBM progression [179]. This raises questions as to whether TAMs induce neutrophil infiltration to tumors similar to what they do during the microbial challenge, or if they have tumor-specific interactions, which warrants further investigation.

In addition to interacting with cells of the adaptive immune system, studies have also demonstrated that TAMs actively interact with other non-immune non-neoplastic cells in the TME. Among the non-immune cells that compose the tumor perivascular niche are endothelial cells, pericytes, and astrocytes [108, 180]. An integral part of the perivascular niche is vigorous and abnormal angiogenesis, leading to disorganized and dysfunctional blood vessels. These blood vessels can evolve into glomeruloid microvascular proliferation (GMP), in which endothelial cells and pericytes form poorly-organized and dysfunctional vascular structures reminiscent of kidney glomeruli [181]. TAMs control tumor angiogenesis by sensing hypoxia conditions, producing IL-1β and increasing VEGFA expression, which are regulators of vascular permeability and tumor angiogenesis [182]. The TAM subset called tunica interna-endothelial cell kinase 2 -Tie-2^+^ cells, found in the perivascular sites in GBM, produce IL-6 and recruit or promote de novo formation of endothelial cells [183]. TAMs also increase endothelial-like markers such as Willebrand factor, CD31, vascular endothelial (VE)-cadherin, and CD105 [184, 185] and cooperate with endothelial cells to form the endothelial lining of tumor blood vessels [186]. In turn, endothelial cells have been found to promote and maintain GSC proliferation by secreting IL-8 [187], while GSCs can trans-differentiate into epithelial cells, promoting tumor growth [188–190]. Pericytes, which surround the endothelial cells, have been shown to regulate immune functions by releasing anti-inflammatory molecules such as IL-10 and TGF-β, or by decreasing immunostimulatory molecules such as MHC II [191]. Astrocytes support GBM survival and invasion by interacting with tumor cells via gap junctions, helping the tumor evade chemotherapy or, in conditions of hypoxia, by releasing cytokines and chemokines that lead to disruption of ECM, thereby facilitating tumor cell invasion [180, 192]. In turn, glioma cells insert themselves between the endfeet of astrocytes and the endothelial wall of blood vessels, which facilitates their invasion and causes focal breach of the BBB, thus permitting molecules and cells from the circulation to enter the CNS even further from the main tumor mass [193]. Heiland et al. recently showed that CD274^+^ reactive astrocytes are enriched specifically in the peritumoral cortex of de novo and recurrent GBM patients. The authors further showed by using an organotypic human brain culture model that the presence of microglia is essential for the increase in reactive and tumor-promoting astrocytes in GBM, and that astrocyte-microglia complex interactions promote immunosuppressive GBM TME [194]. Interactions between astrocytes and microglia in the context of CNS injury and disease have been extensively studied. For example, it has been shown in various human neurodegenerative diseases that activated microglia produce IL-1α, TNF, and C1q, and induce the upregulation of neurotoxic A1 astrocytes that induce the death of neurons and oligodendrocytes [195]. It is plausible that IL-1α-mediated astrocyte-microglia interactions might also play a role in GBM, where neuronal death is high and IL-1 levels are upregulated [3].

Recently, glutamatergic neurons have also been shown to regulate and advance glioma progression by secreting neuroligin-3 (NLGN3) [196, 197]. NLGN3 mediates synaptic communications between glioma cells and neurons through AMPA receptors and the glutamate signalling pathway. Also, a subset of glioma cells has been shown to respond to non-synaptic neuronal activity, reflecting an influx of extracellular potassium, which is a behaviour comparable to normal astrocytes in response to neuronal activity. Gap junction-coupled glioma networks have been observed to support tumor proliferation by amplifying changes in the extracellular ionic compartment [198]. By using electron microscopy, another research group detected a physical coupling between glioma cells and neurons via tumor microtubes (TMs). They defined three morphologically-distinct synaptic contacts responsible for different functional properties, which ultimately support tumor proliferation: i) a single synaptic contact to a glioma TM, ii) a multi-synaptic contact between both a glioma TM and a neuron, and iii) a glioma TM approaching a pre-existing neuronal synapse with contact to the synaptic cleft. By using confocal microscopy, they further identified glutamatergic AMPA receptors to be main players involved in these neuroglioma synaptic networks [199]. Whether TAMs are interacting with neurons in the TME of GBM to promote tumor growth, similar what has been shown in LGGs, will be an interesting novel research area.

**Fig. 4:**
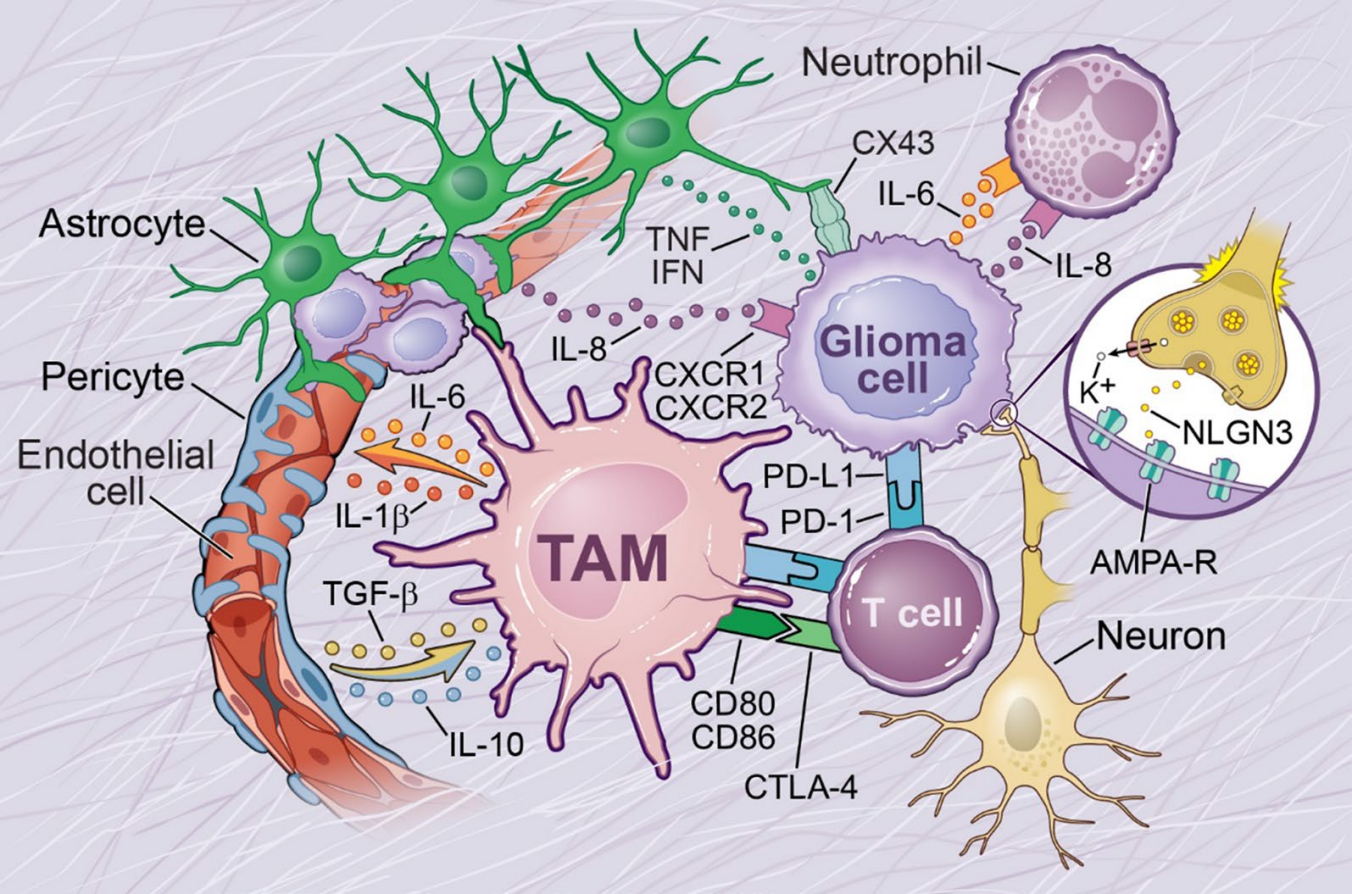
The glioblastoma microenvironment. Schematic illustration of TAM interactions with glioma cells and the non-neoplastic cells of the tumor microenvironment, including endothelial cells, pericytes, activated astrocytes, and T cells. In addition, interactions between tumor cells and neutrophils, neurons, and astrocytes are illustrated. TAMs communicate with the other non-neoplastic cells to regulate tumor progression, either by direct contact or via secreted factors

Overall, these results collectively demonstrate that non-immune non-neoplastic cells in the GBM TME contribute to tumor growth, GSC phenotype, and therapy resistance not only by direct interaction with tumor cells, but also by creating complex interdependency with each other (Fig. 4).

## Macrophage-targeted therapies

There is sufficient evidence to show critical roles for TAMs in GBM growth, invasion, immune evasion, and edema. These studies provided rationale for single-agent or combinatorial TAM-targeted therapies as viable alternatives for GBM treatment. TAM targeting therapies are based upon two strategies: 1) altering their tumor-promoting function, often referred to as re-polarization, and 2) blocking their infiltration.

*Strategies altering TAM function.* The use of CSF1R small-molecule inhibitors, such as BLZ945, has been shown to decrease glioma progression by polarizing TAMs into an anti-tumor phenotype in a PN mouse model of GBM [60]. However, further pre-clinical trials studying the long-term administration of BLZ945 showed that the tumors rebounded rapidly after a dormancy phase of 4 weeks [200]. This resistance was mediated by TAMs via the secretion of insulin growth factor 1 (IGF-1) in the extracellular space after the release of IL-4, probably produced by T cells in response to the drug. IGF-1 interactions with its cognate receptor IGF-1R on the surface of tumor cells and phosphatidylinositol 3-kinase (PI3K) signaling pathway activation resulted in tumor resistance and proliferation [200]. Unfortunately, CSF1R inhibitor as a single agent failed to demonstrate effectiveness in a clinical trial with unselected adult recurrent GBM patients [104].

A recent gene therapy-based approach targeting TAMs is in Phase I/II for treatment of unmethylated O [6]-methylguanine-DNA methyltransferase (MGMT) GBM (NCT03866109). The drug is called Temferon, and it consists of autologous CD34^+^-enriched hematopoietic stem/progenitor cells (HPSCs) transduced with a lentiviral vector that drives the myeloid cells to produce IFN-α, an anti-tumoral cytokine. This strategy provides a promising opportunity for brain tumor therapy, as it successfully inhibited tumor growth in a mouse model of breast cancer [201].

*Strategies blocking TAM recruitment.* The CCL2/CCR2 axis is essential for monocyte recruitment to the tumor site, and genetic reduction of CCL2 levels prolonged the survival of GBM-bearing mice without significant effects on TAM infiltration, which can be partially explained by the redundancies in chemokine functions involved in monocyte recruitment. A few clinical trials are currently ongoing using antagonists for CCL2 and CCR2 to treat solid tumors [202]. Another approach to block TAM recruitment is to target CD47. Two recent ongoing Phase I trials are testing the efficacy of two monoclonal antibodies, IBI 188 (NCT03763149) and SRF-231 (NCT03512340), which are being used as monotherapies in patients with advanced malignant tumors and lymphomas.

Another promising target to block TAM recruitment is the SDF-1 receptor CXCR4. Several CXCR4 antagonists, such as peptide R or LY2510924, have been shown to be beneficial in GBM mouse models [203, 204]; however, they have not moved forward into clinical trials. One additional completed Phase I/II clinical study analyzed the toxicity and efficacy of CXCR4 inhibitor, Plerixafor, in GBM patients after RT and temozolomide (NCT01977677). The Phase I/II study suggested that infusion of Plerixafor was well-tolerated as an adjuvant to chemoradiation in newly-diagnosed GBM patients and improved the local control of tumor recurrence [205].

*Combinatorial therapies.* Considering TAM’s immunosuppressive function and despite failed efficacy of CSF1R inhibitor as a single agent in unselected adult recurrent GBM patients [104], combinatorial therapies targeting CSF1R with novel emerging immunotherapies have been initiated to determine whether there are synergistic effects. One ongoing Phase I trial is currently evaluating the combinatorial effects of the anti-CSF1R antibody, Cabiralizumab, and the anti-PD-1 antibody Nivolumab in patients with advanced malignant glioma (NCT02526017). Another Phase I trial investigates the use of the anti-CSF1R monoclonal antibody SNDX-6352, alone or in combination with an anti-PD-1 antibody, Durvalumab, in patients with solid tumors (NCT03238027).

In addition, oncolytic virotherapies have been proposed to target and reprogram TAMs, in addition to directly killing tumor cells [206]. Recent studies demonstrated that administration of IL-12-loaded nanoparticles in tumor-bearing mice is beneficial for survival and TAM re-education toward an anti-tumorigenic phenotype [207], and resulted in an ongoing Phase I trial in recurrent and progressive GBM patients using Nivolumab together with an adenovirus that induces the production of hIL-12 (NCT03636477).

Additionally, a recent study has shown that there is a CD73^hi^ macrophage population in human GBM that persists after ICI treatment [208]. By using reverse translational studies that exploited the use of CD73^−/−^ mice and syngeneic glioma models, they were able to show that the absence of the molecule significantly improved survival, especially after treatment with PD-1 and CTLA-4 inhibitors, suggesting a new possible target, CD73, to improve the response to therapy ICI [208].

*Several novel concepts and strategies for targeting TAMs are emerging.* Targeting the antigen presentation capacity of TAMs to recruit and (re)-activate anti-tumoral effector T cells is one such option. The use of stimulator of interferon gene (STING) agonists can also be a solution, as STING has shown to increase TAM production of IFN, re-educating TAMs into a pro-inflammatory phenotype, as well as to recruit and activate T cells in murine models of GBM [209] and non-small cell lung cancer [210]. Although these are initial studies, it is tempting to speculate whether the classical professional antigen-presenting cells may be used to enhance the toxic T-cell response in GBM. Continuous effort towards novel ways of TAM re-education into a tumor-suppressor phenotype is another an ongoing strategy. Nucleic acid-based strategies may be a compelling design for future studies. Recent works investigated the role of microRNAs, like miR-142-3p, which targets TGF-β, or let-7b, which activates TLR7 in TAMs, resulting in reduced glioma growth [211, 212]. However, microRNA-based therapy still has the caveat of requiring an effective and safe formulation for in vivo delivery. Another attractive direction is utilization of TAM metabolism, which may provide novel targets. Like tumor cells, TAMs have been shown to switch to aerobic glycolysis, which allows rapid energy generation during hypoxia conditions, mediated by the AKT-mToR-HIF-1α pathway. Increased glycolysis results in increased lactic acid production, which ultimately induces TAMs to express tumor suppressor factors such as ARG1 and VEGFA [213].

All of these therapies are designed to target TAMs as a whole, but as we have discussed in this review, there are essential differences in ontogeny, regional heterogeneity, and localization of microglia and macrophages in GBM that might result in their differential functions. In addition, with novel discoveries demonstrating that various genetic driver mutations can create different microglia/macrophage composition in tumors, it is becoming apparent that we need more research to further discriminate between the biological functions of microglia versus macrophages in GBM. As a field, we have not yet explicitly succeeded in depleting one versus the other, first to demonstrate whether there is a redundancy in function between microglia and macrophages, and also what any differences might be. Understanding these differences will open novel avenues of specific TAM sub-population targeting therapies, which could be tailored to tumor genotype or subtype.

## Conclusions

**Fig. 5:**
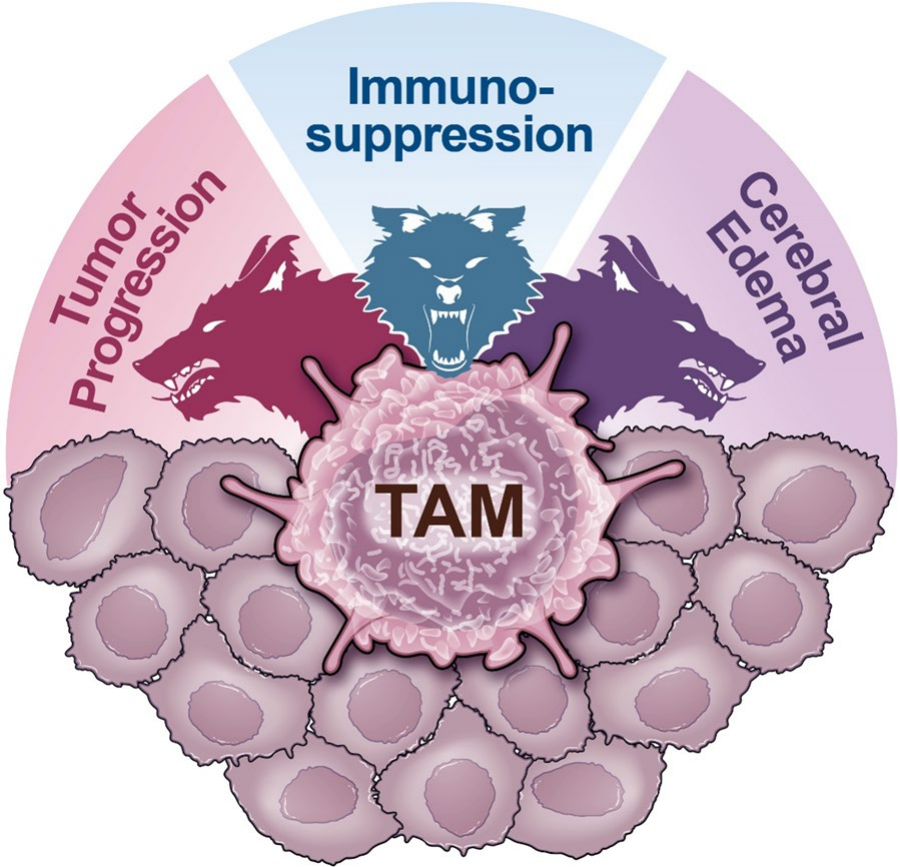
Tumor-associated macrophages (TAMs): The Cerberus of glioblastoma (GBM). Representation of the main functions of TAMs in promoting GBM, including contributions to tumor progression, immunosuppression, and development of vasogenic cerebral edema. TAMs can be metaphorically compared to the three-headed dog Cerberus from Greek mythology, who guards the Underworld gates, preventing the dead from living. In GBM, TAMs protect the tumor cells from dying and support their living

High-grade gliomas, such as GBM, have high degrees of inter- and intra-tumor heterogeneity, with no effective therapy to date. Despite several pre-clinical and clinical trials being conducted, beneficial outcomes have been very limited. Recently, it has become clear that TAMs, including tissue-resident microglia and invading BMDMs, play critical roles in GBM growth, invasion, immune evasion, and resistance to therapy. TAMs contribute to the high levels of tumor heterogeneity and malignancy by playing a triple tumor-supporting role that can be easily compared to the mythological evil three-headed dog, Cerberus; these myeloid cells support tumor proliferation, regulate immunosuppression, and contribute to cerebral edema (Fig. 5). Each of these functions are interconnected, and to date, are still not fully understood. In a way, TAMs protect the tumor just as Cerberus guards the underworld.

Although genetically stable, TAMs are very plastic cells for their ability to change their expression profile according to specific signals and interactions, which results in highly heterogeneous TAM populations in GBM. Therefore, deep immune profiling at the transcriptional and protein levels in human resected brain tumor samples will contribute to improving our understanding of this intricate puzzle of TAM heterogeneity. It will help in defining specific functional roles of TAMs in spatiotemporal organization, and it also may provide distinct TAM signatures for microglia and macrophages. Additionally, as increasing evidence shows that GBM subtypes dictate the tumor cellular components, as well as the response to therapy, subtype discrimination is one of the main parameters to consider for future studies, especially in the context of IDH mutation. However, given the fact that spatial cellular organization and heterogeneity are important in GBM, surgical tissue resection represents a limiting factor in defining the real cellular composition in human tissues. This challenge can be partially overcome by using a high number of samples, decreasing the variance, and increasing the tissue coverage, and possibly by evaluating DEX-naïve samples. Another interesting aspect to elucidate would be the role of BAMs in the onset and malignancy of GBM. Whether the different ontogeny and cell development may play a role in tumor progression can be speculated. Once specific aberrantly-expressed TAM markers are defined, whether they are novel or were identified previously, it is important to develop proper and efficient mouse models to study the molecular pathways and to start pre-clinical trials. Elucidating other molecular mechanisms involved in glioma-associated cerebral edema, besides the IL-1 pathway, is another notable strategy to find a more optimal anti-edema therapy.

Clearly, communication between TAMs and not only tumor cells, but also the TME, cannot be underestimated and requires further investigation. Finally, complementary studies, and perhaps multi-targeted therapies, need to be validated for efficiency and side effects in combination with classical therapies in multiple models to cover GBM heterogeneity. Considering all of these aspects, future research should attempt to exploit the potential of TAMs for the development of beneficial therapies against brain tumors, and particularly GBM.

## BOX 1

### Glioblastoma: inter- and intra-tumor heterogeneity

Glioblastoma (GBM) is an incurable malignancy, despite aggressive treatment regimens. The standard of care for GBM consists of maximal surgical resection followed by radiation and concomitant and maintenance temozolomide (TMZ) administration [214]. The median survival time of GBM patients is only ∼15 months [214], with ~ 70% of recurrences within one year and ~ 5% survival after 5 years [215]. These tumors are chemo- and radiotherapy resistant [216]. GBM resistance can be partially attributed to the infiltrative nature of GBM cells, which makes surgical resection incomplete; remaining malignant cells in the post-surgical cavity inevitably lead to tumor regrowth, usually within 2–3 cm from the border of surgical resection [217].

An additional contributing factor for GBM resistance is the heterogeneity of these tumors within neoplastic cells, consisting of both inter- and intra-tumor heterogeneity [218, 219]. Inter-tumor heterogeneity refers to GBM from different patients with altered genotype and phenotype, while intra-tumor heterogeneity refers to genomic and biological variations within the same tumor, at both the regional and single-cell levels. Over a decade ago, The Cancer Genome Atlas (TCGA) initiative provided robust gene expression-based identification of GBM subtypes, including Proneural (PN), Mesenchymal (MES), and Classical (CL) [55, 220–222]. These subtypes were established based upon the dominant transcriptional patterns at the time and location of tumor resection, so they are not mutually exclusive (i.e. multiple subtypes can co-exist within a single tumor, both at the regional [223] and single-cell levels [224]). Additionally, using multiple high-dimensional approaches with the goal of defining a unified model of cellular states and genetic diversity, one study found that malignant cells in GBM exist in four main cellular states, which closely resemble distinct neural cell types, are affected by the tumor microenvironment (TME), and most importantly, are plastic [225].

It was also documented that amplification and/or mutation of the *epidermal growth factor receptor (EGFR)* is predominant in CL GBM, whereas *neurofibromin 1* (*NF1)* deletions are frequent in MES GBM and platelet-derived growth factor receptor alpha (*PDGFRA)* gene amplification is common in PN GBM [55, 64, 222], although none of these are mutually exclusive. These studies resulted in multiple classification strategies, but in 2016 the WHO definitively classified GBM by using both histological and molecular genetic features [226]. In particular, this new classification system divides GBM into *isocitrate hydrogenase-wildtype (IDH WT)*, where IDH is not mutated. This represents ~ 90% of the cases and occurs more frequently as primary or de novo tumors in patients over 55 years of age. *IDH-mutant* (*IDH MUT*) GBM represents the remaining ~ 10% of cases and usually arise as secondary tumors in younger people. They are associated with significantly better overall survival compared to patients with *IDH WT* tumors [226].

## BOX 2

### Glioma stem cells

Regardless of the cell of origin, many cancers are defined hierarchical structures, in the apex of which Glioma Stem Cells (GSCs) reside. These cells exhibit unique properties, self-renewal, multipotency, and have increased tumor initiation ability upon transplantation into recipient animals. They are considered to be a relatively rare population in tumors, which give rise to more-differentiated non-glioma stem cells that constitute a large proportion of tumor mass. In contrast to GSCs, non-glioma stem cells have a significantly lower ability to initiate tumors upon transplantation into recipient animals [227, 228]. However, GSC characterization is still quite challenging to date [227]. Several markers have been proposed to define this cell population, such as CD133, CD44, SSEA1, and CD49f among others [229–232], which are more resistant to chemo- and radiation therapy and are believed to be responsible for tumor recurrence (more details on GSC biology are provided in [1, 4]).
